# A novel MPPT design based on the seagull optimization algοrithm for phοtovοltaic systems operating under partial shading

**DOI:** 10.1038/s41598-022-26284-x

**Published:** 2022-12-16

**Authors:** Abdelilah Chalh, Redouane chaibi, Aboubakr El Hammoumi, Saad Motahhir, Abdelaziz El Ghzizal, Mujahed Al-Dhaifallah

**Affiliations:** 1 Innovative Technologies Laboratory, Higher School of Technologies, SMBA University, 30000 Fez, Morocco; 2 Industrial Technologies and Services Laboratory, Higher School of Technologies, SMBA University, Fez, Morocco; 3 Engineering, Systems and Applications Laboratory, ENSA, SMBA University, 30000 Fez, Morocco; 4 Control & Instrumentation Engineering Department, King Fahd University of Petroleum & Minerals, Dhahran, 31261 Kingdom of Saudi Arabia; 5 Interdisciplinary Research Center (lRC) for Renewable Energy and Power Systems, King Fahd University of Petroleum & Minerals, Dhahran, 31261 Kingdom of Saudi Arabia

**Keywords:** Engineering, Electrical and electronic engineering

## Abstract

The use of a maximum power point (MPP) tracking (MPPT) controller is required for photovoltaic (PV) systems to extract maximum power from PV panels. However, under partial shading conditions, the PV cells/panels do not receive uniform insolation due to several power maxima appear on the PV array's P–V characteristic, a global MPP (GMPP) and two or more local MPPs (LMPPs). In this scenerio, conventional MPPT methods, including pertub and observe (P&O) and incremental conductance (INC), fail to differentiate between a GMPP and a LMPP, as they converge on the MPP that makes contact first, which in most cases is one of the LMPPs. This results in considerable energy loss. To address this issue, this paper introduces a new MPPT method based on the Seagull Optimization Algorithm (SOA) to operate PV systems at GMPP with high efficiency. The SOA is a new member of the bio-inspired algorithms. When compared to other evolutionary techniques, it uses fewer operators and modification parameters, which is advantageous when considering the rapid design process. In this paper, the SOA-based MPPT scheme is first proposed and then implemented for an 80 W PV system using the MATLAB/SIMULINK environment. The effectiveness of the SOA based MPPT method is verified by comparing its performance with P& O and PSO (particle swarm optimization) based MPPT methods under different shading scenarios. The results demonstrated that the SOA based MPPT method performs better in terms of tracking accuracy and efficiency.

## Introduction

In several nations, photovoltaic (PV) power systems are widely employed. However, several urgent challenges must be addressed in the deployment of these systems. One of the biggest issues is how to increase efficiency^1–3^. Under uniform irradiation conditions, i.e. when PV cells/panels receive uniform insolation, the PV array’s P–V characteristic exhibits a single power maxima, which is known as the maximum power point (MPP). Therefore, tracking this MPP is crucial in a PV system to optimize the power output of the PV array. Moreover, as the power withdrawn from the PV array is strongly affected by cell temperature, irradiance, and load impedance^4,5^, PV systems must be designed to operate at MPP regardless of the variation of these factors.

Several MPPT strategies, including perturb and observe (P&O)^6–9^ and incremental conductance (INC)^10–12^, have been suggested to improve PV system performance. In particular, the P&O approach employs a disturbance in the PV system's operational voltage^7^. However, the presence of oscillations around the MPP, as well as its restricted capacity to follow this point under transitory environmental conditions, are the key drawbacks of this strategy. The INC strategy^10^ was proposed to decrease these oscillations and enhance system efficiency, but it did not totally eliminate oscillations. Furthermore, these systems employ a set step to determine the ideal duty cycle value, which may result in incorrect or sluggish tracking during abrupt changes in temperature or irradiance^11^. Fuzzy logic, neural networks, and neuro-fuzzy are examples of intelligent algorithms. These algorithms use a variable step to determine the ideal duty cycle value, resulting in a quicker time response and greater stability under varied operating circumstances.

However, PV arrays are frequently subjected to partial shading conditions (PSCs), which are the root cause of the majority of output power decrease and mismatch^13^. When the PV array is operating under these conditions, the P–V curves are characterized by the appearance of many local peaks, which are caused by the activation of bypass diodes, which protect shaded cells^14^. In such partial shading conditions, standard MPPT algorithms may miss the target by converging to a local maximum rather than the global maximum, resulting in a large loss in output power and, as a result, a poor overall system yield. A variety of enhancements to traditional MPPT algorithms have been developed to deal with the impact of shading on the P–V curves. Some are topology-based and require extra power circuits to accomplish global MPPT (GMPPT)^15^. As a result, overall efficiency is lowered. Others are algorithm-based strategies such as fuzzy logic with polar controller and sequential extremum searching control^16^. The effectiveness of soft computing methods in handling nonlinear problems, such that encountered in PV array behaviour, and their implementation simplicity make them very attractive to solve the MPPT problem of PV systems, especially in the case of partial shading and module mismatches^17^. Artificial Neural Networks, are one of soft computing methods that was used in MPPT techniques. Typically, they were used to estimate the MPP with respect to the randomly changing weather conditions^18^, and to improve the P&O and IC algorithms^19^. These approaches are expensive, time-consuming operations that necessitate the use of complicated technology. However, this solution can increase the cost of the PV system due to the high number of used sensors. Evolutionary computation techniques, such as Differential Evolution (DE)^20^, has been also proposed to deal with the MPPT problem. However, EC techniques might present a poor convergence rate and slow convergence time^21,22^. The metaheuristics techniques have a good convergence rate and fast convergence compared to EC techniques. In addition, the application of the metaheuristic algorithm for MPPT has attracted the interest of many researchers due to its ability to handle nonlinear functions without requiring derivative information. Since metaheuristic MPPT approaches are an efficient search and optimization method for real-valued multi-modal objective functions, it is envisaged to be very effective to deal with MPPT problems. Various metaheuristic approaches are found in the literature but the more popular ones are particle swarm optimization (PSO), grey wolf optimization (GWO), ant colony (ACO), Artificial Bee Colony (ABC), Whales Optimization Algorithm (WOA)^23–28^. Sarvi et al. in^29^ proposed the PSO-based MPPT for PV systems under PSC to find the GMPP. Nevertheless, this solution presented oscillations around the steady-state. Hence, some researchers have attempted to improve the PSO to reduce oscillations^30,31^. However, their improved method cannot follow the dynamic GMPP under various shading patterns. Furthermore, Jang et al. in^32^ proposed an ACO algorithm and showed that this method has a faster convergence speed compared to the Basic PSO. ACO and PSO methods present a major disadvantage in terms of convergence linked to the initial placement of the agents into the research space. In addition, both PSO and ACO need the determination of many parameters, making them rigid and complicated.

To overcome these complexities found in the PSO and ACO methods, the authors of^33^ established a comprehensive bio-inspired approach for addressing computationally costly issues called the seagull optimization algorithm (SOA), which mimics the search and attack behaviors of seagulls in nature. This algorithm is one of the latest effective optimization methods, which is gradient-free and applicable to optimize all engineering problems occurring in real life. Additionally, compared to other evolutionary algorithms, SOA requires fewer variables for adjustment and fewer operators, which is advantageous when considering a speedy design process^33^. This algorithm is divided into two phases: the exploration and exploitation phases. During the exploration phases, the search agent makes larger update steps to the candidate solutions. On the other hand, the search agents seek to make use of the search process's history and experience during exploitation. In^34^, the authors present a Modified Seagull Optimization Algorithm (MSOA) based MPPT approach by incorporating Levy Flight Mechanism (LFM) and the formula for heat exchange in Thermal Exchange Optimization (TEO) into the original Seagull Optimization Algorithm (SOA). Thus, in this article, their results from the simulation of the exploration phase is not clear. In which it is calculating the fitness values of each search. Yet, to the best of the authors' knowledge, no research has been done on MPPT based on SOA so far, which motivates us to study this method and to enrich the scientific references with the developed version of the original SOA for MPPT controllers. To this end, this work proposes an SOA-based metaheuristic MPPT method for tracking the GMPP to maximize the PV power output in PV systems operating under both uniform and partial shading conditions. This method is considered best suited for real engineering problems compared to another metaheuristic algorithm. The MPPT's speed and efficiency will be considerably improved.

After the introduction, in "The effect of shading on PV array" section  briefly presents the effect of partial shading on the PV array characteristics. ïn Section ″Selection the parameters of Boost converter″ introduces the SOA’s fundamentals and mathematical model. in Section ″Seagull optimization algorithm (SOA)″ presents the proposed MPPT controller and how it developed based on SOA. in Section ″Results and discussion″ gives and discusses the simulations results of the proposed SOA-based MPPT method, along with a comparison of its performance with PSO and P&O based MPPT methods. Finally, "Processor In the Loop (PIL) testing" section summarizes the results and suggests some recommendations for further research.

## The effect of shading on PV array

### PV cell and module modeling

**Figure 1:**
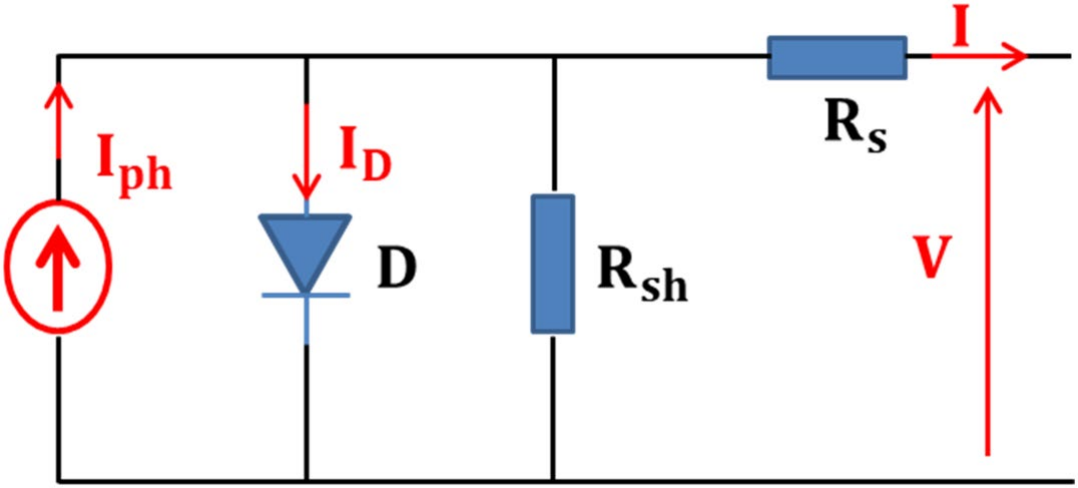
The equivalent circuit for the single diode mοdel οf the solar cell.

The electrical model of the PV cell, as illustrated in Fig. 1, consists of a current source, a diode, and a resistor Rsh linked in parallel, as well as a series resistor Rs.

Where the current source is proportional to sun irradiation. The Rs is primarily determined by the metal base's contact resistance with the p semiconductor layer, the p and n bodies' resistances, the n layer's contact resistance with the top metal grid, and the grid's resistance. The Rsh resistance is mostly influenced by the leakage current of the p–n junction and is affected by the PV cell production procedure^35^.

Based on the Fig. 1, the following equation^35^ gives the output current:

1 $$I = I_{ph} - I_{0} \left( {exp\frac{{q\left( {V + IR_{s} } \right)}}{{AV_{t} }} - 1} \right) - \frac{{V + IR_{s} }}{{R_{sh} }}$$

where $I_{ph}$*,* $I$*,* and $I_{0}$ are denoted the phοtocurrent, the οutput cell current, and the reverse saturatiοn current respectively. $V$ represents the οutput cell vοltage. $R_{s}$ and $R_{sh}$ present a series resistance and parallel resistance respectively.$A$ is the diοde ideality factor.

The value of the termal voltage is given by (2).

2 $$V_{t} = \frac{KT}{q}$$

where $K$*,* $q$*,* and $T$ are denoted the Bοltzmann cοnstant (1.38 × 10^−**23**^ (J/K)), the charge of electrοn (1.6 × 10^−**19**^ (C)), and the sοlar cell temperature (K) respectively.

The photocurrent $I_{ph,c}$ of a solar cell depends on many material characteristics. However, it can be approximated as linear-dependent on irradiance and temperature with sufficient accuracy as follow^36^:

3 $$I_{ph,c} = \frac{G}{{G_{ref} }}\left[ {I_{sc,ref} + \mu_{sc} \left( {T - T_{ref} } \right)} \right]$$

where $I_{sc,ref}$ is solar cell short-circuit current at standard test conditions (STC): *G*_*ref*_ = 1000 W/m^2^, *T*_*ref*_ = 25 $^\circ C$.

$\mu_{sc}$ is the solar cell short-circuit temperature coefficient, normally provided by the manufacturer (A/K).

G is the actual irradiance intensity (W/m^2^);

The well-known diode saturation current estimation equation is given by^36^:

4 $$I_{0} = I_{0,ref} \left( {\frac{{T_{ref} }}{T}} \right)^{3} exp\left[ {\frac{{qE_{g} }}{nk}\left( {\frac{1}{{T_{ref} }} - \frac{1}{T}} \right)} \right]$$

where, the nominal saturation current *I*_*0,ref*_ at STC is given by:

5 $$I_{0,ref} = \frac{{I_{sc,ref} }}{{exp\left( {\left( {V_{oc,ref} /nV_{t} } \right) - 1} \right)}}$$

*V*_*oc,ref*_ is solar cell open-circuit voltage at reference condition.

*E*_*g*_ is band-gap energy in the solar cell, (1.12–1.15 eV).

To achieve the desired voltage and current levels, Ns cells are connected in series and NP cells are connected in parallel respectively, thus forming a PV module. There for the PV module parameters are scaled according to NS and NP as given bellow^37^:

6 $$I_{ph\_Total} = N_{p} *I_{ph,c}$$

7 $$I_{0\_Total} = N_{p} *I_{0}$$

8 $$A_{Total} = N_{s} *A$$

9 $$R_{s\_Total} = \frac{{N_{s} }}{{N_{p} }}*R_{s,c}$$

10 $$R_{sh\_Total} = \frac{{N_{s} }}{{N_{p} }}*R_{sh,c}$$

**Figure 2:**
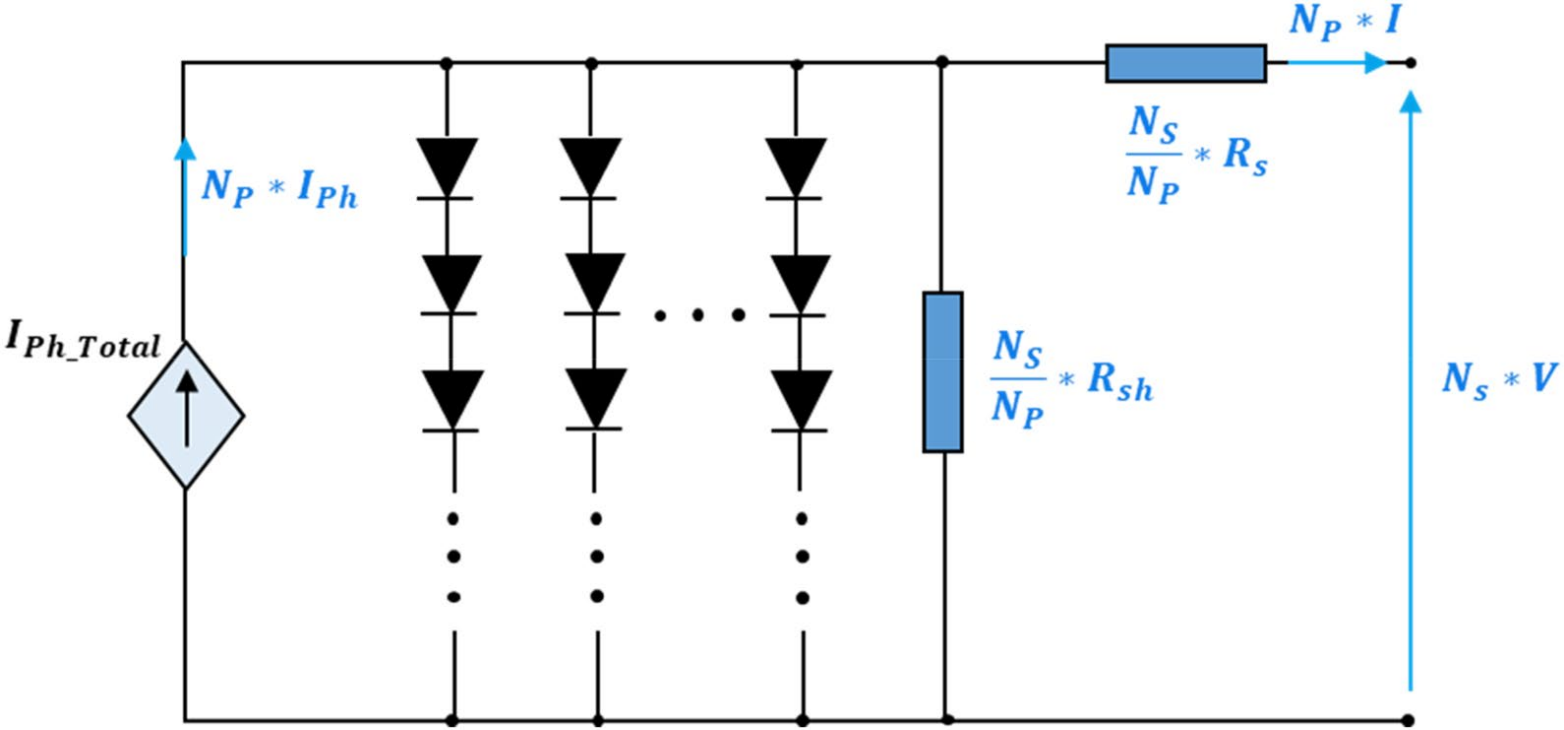
The equivalent circuit of the PV module.

The overall PV module model can then be represented by the following Fig. 2.

Current–voltage characteristic equation of equivalent circuit for the PV module arranged in Np parallel and Ns series cells is given by^32^:

11 $$I^{M} = N_{p} I_{ph} - N_{p} I_{0} \left( {exp\frac{{q\left( {V^{M} /N_{s} + I^{M} R_{s} /N_{p} } \right)}}{{AV_{t} }} - 1} \right) - \frac{{\left( {\left( {N_{p} /N_{s} } \right)V^{M} + I^{M} R_{s} } \right)}}{{R_{sh} }}$$

The characteristics of the PV panel used in this work are shown in Table 1.

**Table 1:** Characteristics of the PV panel TDC**-**M20-36 at STC^38^.

TDC-M20-36 PV panel at STC	
Maximum power	20 W
Maximum voltage	18.76 V
Maximum current	1.07 A
Short-circuit current	1.17 A
Open-circuit voltage	22.70 V
Temperature coefficient of open-circuit voltage	− 0.35%/°C
Temperature coefficient of short-circuit current	− 0.043%/°C
Number of cells	36
Type of cells	Monocrystalline

### Effect of partial shading on PV array

**Figure 3:**
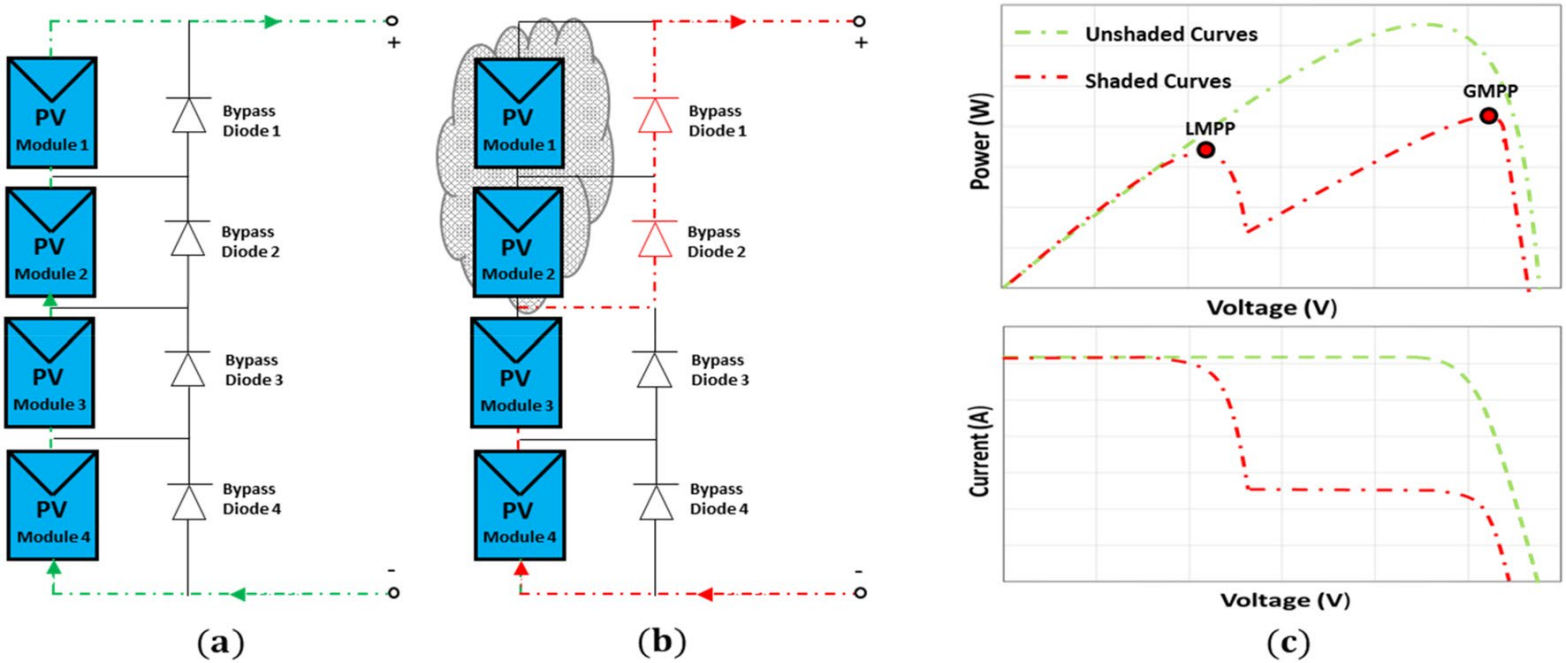
PV array functionality: (**a**) Unshaded condition of PV array, (**b**) shaded condition of PV array, and (**c**) I**–**V and P**–**V curves resulting from different scenarios (**a**) and (**b**).

When PV cells (or modules) are partially shaded, they function as a load on other cells/modules and become reverse biased. As a result, instead of generating energy, they will dissipate it, resulting in a rise in cell temperature. The cell/module can be damaged and influence the entire PV module/array if the temperature becomes too high, which is called the hot spot issue. One of the most prevalent methods to avoid the hot spot problem is to connect a bypass diode to a set of cells connected in series^38,39^, as illustrated in Fig. 3.

To apprehend the current flow direction of the PV array under PSC, consider the PV array in Fig. 3. The PV array consists of four PV modules where two PV panels are unshaded and the other are shaded, as illustrated in Fig. 3b. The P–V curve of the PV array under PSC can be divided into two phases. During uniform solar irradiance, the bypass diodes are reverse biased and therefore have no effect (Fig. 3a). In the other phase (Under PSCs), when the load current is higher than the shaded PV module, the bypass diode active. But, when the load current is lower than the shaded PV module, the bypass diode stays inactive as can be seen in Fig. 3b.

## Selection the parameters of boost converter

**Figure 4:**
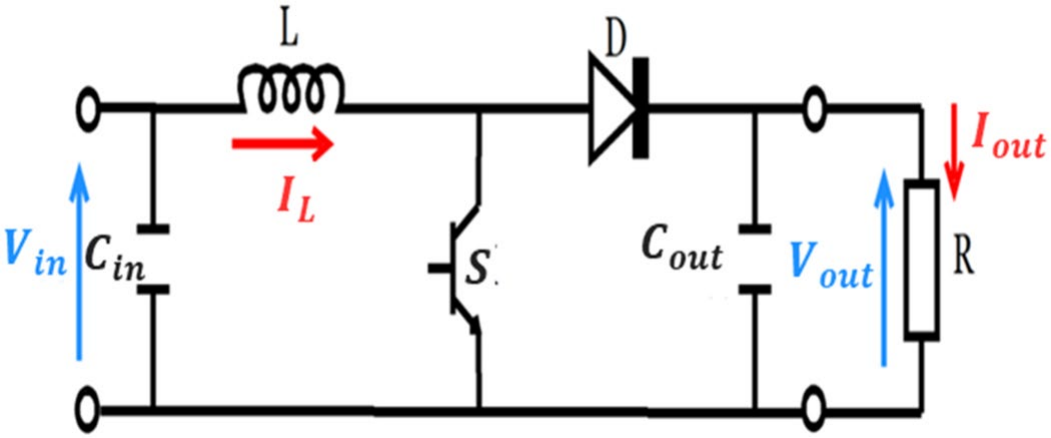
DC**–**DC converter.

A boost converter (step-up converter) is a power converter with an output DC voltage greater than its input DC voltage^41^. It is a class of switching-mode power supply (SMPS). A simple boost converter consists of an inductor L, a controlled switch S and a diode D, filters made of a capacitors are normally added to the output and the input of the converter to reduce voltage ripples (see Fig. 4).

### Inductor selection of boost converter

Estimate the inductor ripple current is 20% to 40% :

12 $$\Delta I_{L} = I_{out\_\max } \times \left( {0.2 to 0.4} \right) \times \frac{{V_{out} }}{{V_{in} }}$$

The critical inductance value of the boost converter is given by the Eq. (13):

13 $$L = \frac{{V_{in} \left( {V_{out} - V_{in} } \right)}}{{\Delta I_{L} \times f_{sw} \times V_{out} }}$$

where:

- $V_{{in{ }}}$ is the input voltage;
- $V_{{out{ }}}$ is the desired output voltage;
- $f_{sw}$ is the designed switching frequency;
- $\Delta I_{L}$ is inductor ripple current;

### Output capacitor selection of boost converter

The current store to the output circuit is discontinuous. Therefore, to limit the output voltage ripple must use a big filter capacitor. When the diode is off, The filter capacitor should supply the output DC to the load.

14 $$C_{out\_\min } = \frac{{D \times I_{out\_\max } }}{{\Delta I_{out} \times f_{sw} }}$$

where:

- $C_{out\_min}$ is the output capacitance(minimum);
- $\Delta V_{out}$ is the ripple of output voltage;
- $f_{sw}$ is switching frequency in kHz;
- $I_{out\_Max}$ is maximum output current;
- $D$ is the duty cycle;

The selection of $C_{min} { }$ must be higher than the calculated value to make sure that the converter’s output voltage ripple remains within the specific range and its equivalent series resistance (ESR) should be low. ESR can be minimized by connecting many capacitors in parallel. Therefore, it can be assumed that the ESR is as in Eq. (15):

15 $$\Delta V_{out\_ESR} = ESR \times \left( {\frac{{I_{out\_\max } }}{1 - D} + \frac{{\Delta I_{L} }}{2}} \right) \approx 0.05 V_{out}$$

### Intput capacitor selection of boost converter

As stated earlier, the output of the PV voltage has ripples due to the change in temperature and irradiation. Therefore, it is necessary to replace the input capacitor in parallel with the voltage supply to minimize ripples produced by the solar panel. The ripples have an adverse effect on the output as the input voltage is proportional to the output current. Similarly to the output capacitor, $ESR$ on the input capacitor should be considered by selecting a greater capacitor value than the calculated one. Equation (16) computes the value of the input capacitor while considering the ripples limit.

16 $$C_{in} = \frac{{\Delta I_{L} }}{{8 \times f_{sw} \times \Delta V_{in} }}$$

where:

17 $$\Delta V_{in} = ESR \times V_{in} \approx 0.05 V_{in}$$

The electrical parameters of the used boost converter are depicted in Table 2.

**Table 2:** The electrical parameters of the used boost converter.

Components	Values
*Inductor, L*	15 mH
Input Capacitοr, C_IN_	22 µF
Output Capacitοr, C_Out_	22 µF
Switching Frequency, f	10 kHz
Load, R_***L***_	220Ω

## Seagull optimization algorithm (SOA)

### The basics of the SOA

Seagulls are a type of coastal bird that has been around for roughly thirty million years. Their wings are large, and their rear legs have developed to enable them to travel on the water. Seagulls come in a range of sizes and shapes, and they may be found in practically every corner of the world. Seagulls are capable of drinking both fresh and saltwater. Most animals are unable to do this. On the other hand, Seagulls have a unique set of drums covering their eyes that they used to clean the salt out of their system by opening their beaks. Seagulls inhabit in vast groups and use a variety of voices to communicate with one another. With their expertise, they can find and attack the prey. They steal food under the influence of other birds, animals, and even people, which is one of their strangest behaviours. Seagulls eat mostly fish, although they also eat earthworms and insects. To discover and attack prey, seagulls use their intelligence. The most prominent characteristics of seagulls are their migratory and attacking habits. A group of seagulls migrated from one area to another using mathematical models of predator movement and attack. A seagull must satisfy the following requirements:

The migration behaviour is described as follows:

- They move in groups when migrating. To avoid accidents, their starting locations differ from one another.
- They use their swarm experience to their benefit that is they try to go in the way of the highest survival to acquire the lowest cost value.

**Figure 5:**
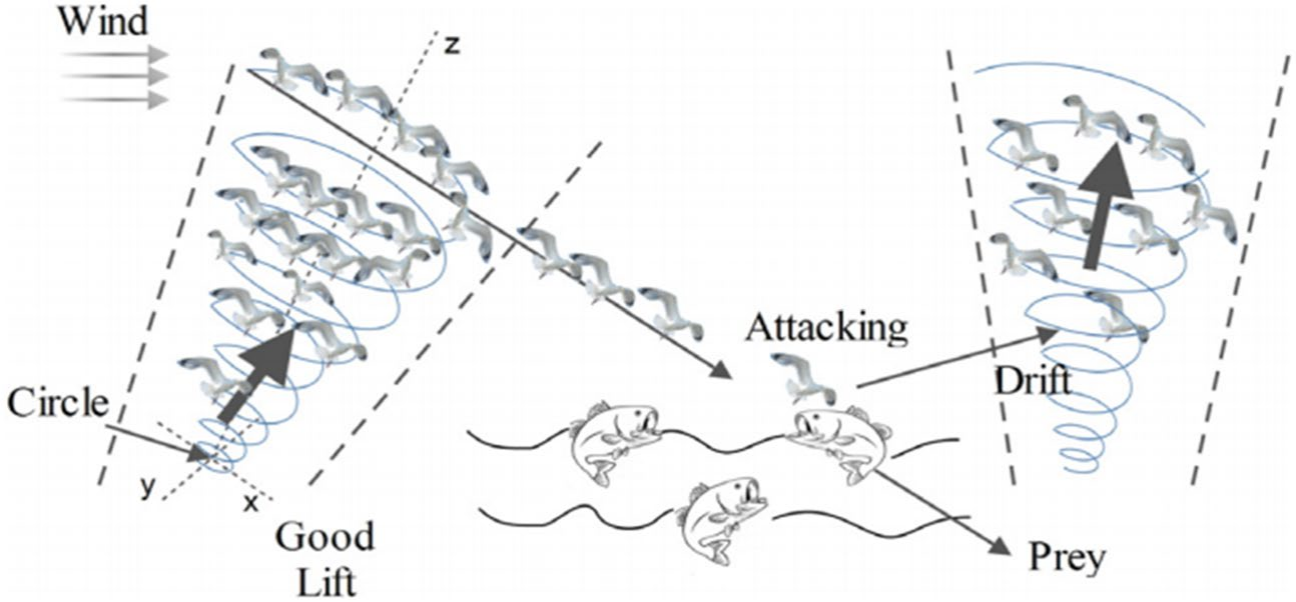
Migration and attacking behaviors of seagulls^33^.

Seagulls typically attack migratory birds over the sea. This procedure is influenced by the natural structure of the spiral's activity during the attack. Figure 5 depicts a conceptual model of these characteristics. The Seagull Model for the Seagull Optimization Algorithm (SOA) is explained more below.

### Mathematical model of SOA

Exploration (migration) and exploitation are the foundations of the SOA's mathematical model (attacking the prey).

During exploration, the algorithm must satisfy three conditions (avoiding collisions, move in the direction of the best neighbour, and stay close to the best search agent) to replicate how a group of seagulls moves from one place to another. The behaviour of the migration can be modelled by the following equation^33^:

18 $$\overrightarrow {{D_{s} }} = \left| {A \times \overrightarrow {{X_{s} }} \left( t \right) + B \times (\overrightarrow {{X_{bs} }} \left( t \right) - \overrightarrow {{X_{s} }} \left( t \right)} \right|$$

19 $$A = fc - \left( {iter \times \left( {\frac{fc}{{Max\_iter}}} \right)} \right)$$

20 $$B = 2 \times A^{2} \times rand$$

where the distance between the current search agent and the best-fit search agent is provided by ${\vec{\text{D}}}_{s}$, ${\vec{\text{X}}}_{{\text{s}}} \left( t \right)$ denotes the current place of search agent, ${\vec{\text{X}}}_{{{\text{bs}}}} \left( t \right)$ denotes the place of the best-fit search agent. $t$ denotes the current iteration, A presents a linearly decreases from fc to 0, B is a randomized variable that ensures a correct balance of exploration and exploitation.

During exploitation, seagulls seek to make use of the search process's history and experience. In this phase, seagulls use their wings and weight to keep their height. During the iteration process, the search agents might update their locations about the best search agent. As a result, the following equation is used to determine the search agent's updated position^33^:

21 $$\overrightarrow {{X_{s} }} \left( {t + 1} \right) = \left( {\overrightarrow {{D_{s} }} \times X^{\prime} \times Y^{\prime} \times Z^{\prime}} \right) + \overrightarrow {{X_{bs} }} \left( t \right)$$

where $\vec{X}_{s} \left( {t + 1} \right)$ represents the equation of updating the position of other search agents. $X^{\prime}$,$Y^{\prime}$ and $Z^{\prime}$ described the spiral movement that behavior produces in the air, and which are defined as follows^33^:

22 $$k = pi.{\text{rand}}$$

23 $$r = u.e^{kv}$$

24 $$X^{\prime} = r.\cos \left( k \right)$$

25 $$Y^{\prime} = r.\sin \left( k \right)$$

26 $$Z^{\prime} = r.k$$

The radius of each spiral turn is r, while k is a random value in [0, 2π]. e is the natural logarithm's base, while u and v are constants that determine the spiral form.

Here is the detailed pseudo-code of the SOA algorithm^33^:
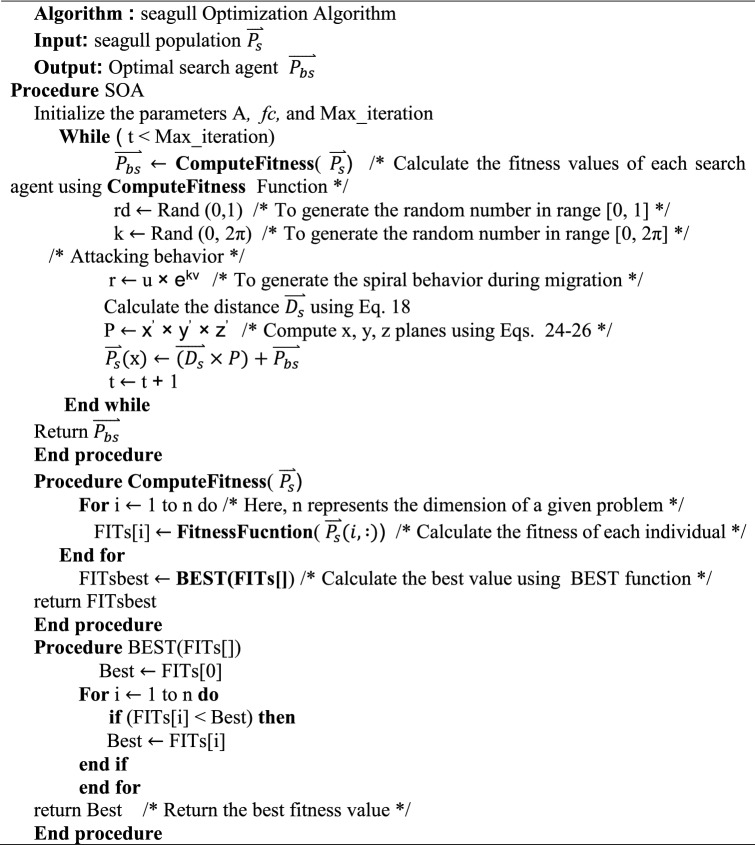


### Application of SOA to the MPPT problem

Each seagull's solution is specified as the duty cycle value of the DC–DC converter to achieve the direct control SOA-based MPPT. In the first iteration, the duty cycle value can be generated using the following equation:

27 $$d_{i} = d_{min} + rand\left[ {0,1} \right]\left( {d_{max} - d_{min} } \right) ; i = 1,2, \ldots N_{SOA} )$$

where $d_{min}$ and $d_{max}$ represent the limit of the search band mechanism.

The distance D_s_ can be calculated using the following equation :

28 $$D_{s} = \left| {A \times d_{i} \left( t \right) + B \times \left( {d_{best} \left( t \right) - d_{i} \left( t \right)} \right)} \right|$$

The new seagull’s solution can then be generated using the following equation:

29 $$d_{new} \left( {t + 1} \right) = d_{best} \left( t \right) + \left( {D_{s} \times X^{\prime} \times Y^{\prime} \times Z^{\prime}} \right)$$

Since the GMPP changes continuously as the weather conditions change, the SOA-MPPT algorithm must be restarted to search for the new GMPP. Therefore, to detect if ever a change in weather conditions takes place to restart the search, the following inequality is adopted in the algorithm:

30 $$\frac{{\left| {P_{{Pv_{new} }} - P_{{Pv_{last} }} } \right|}}{{P_{{Pv_{last} }} }} \ge \Delta P_{Pv} \left( \% \right)$$

Whenever the inequality indicated above is met, the process of finding a new MPP will be repeated to ensure that the algorithm can always identify the GPPM regardless of the operating condition.

**Figure 6:**
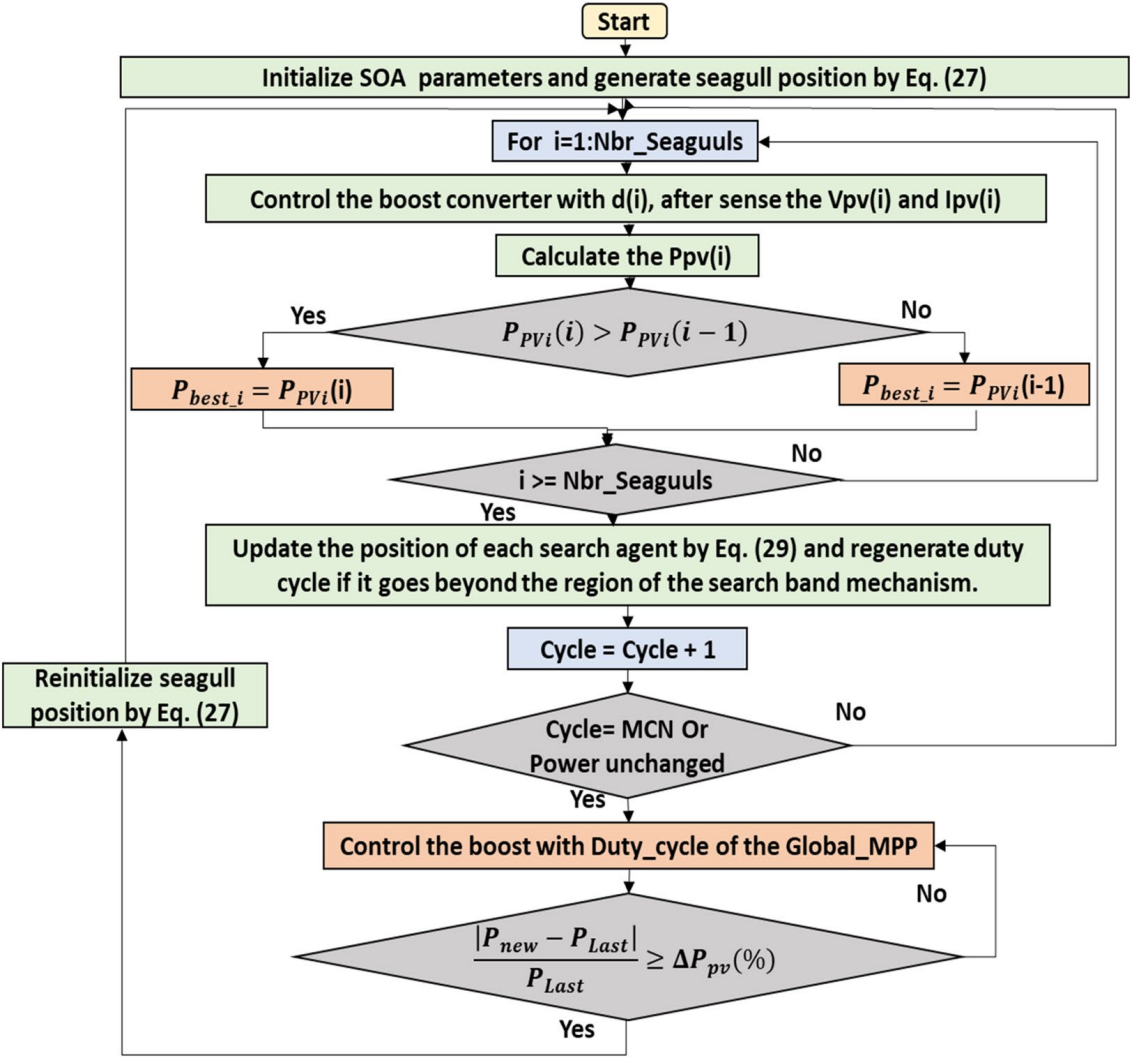
Flowchart of SOA based MPPT algorithm.

Figure 6 depicts the principle working of the SOA based MPPT algorithm.

## Results and discussion

**Figure 7:**
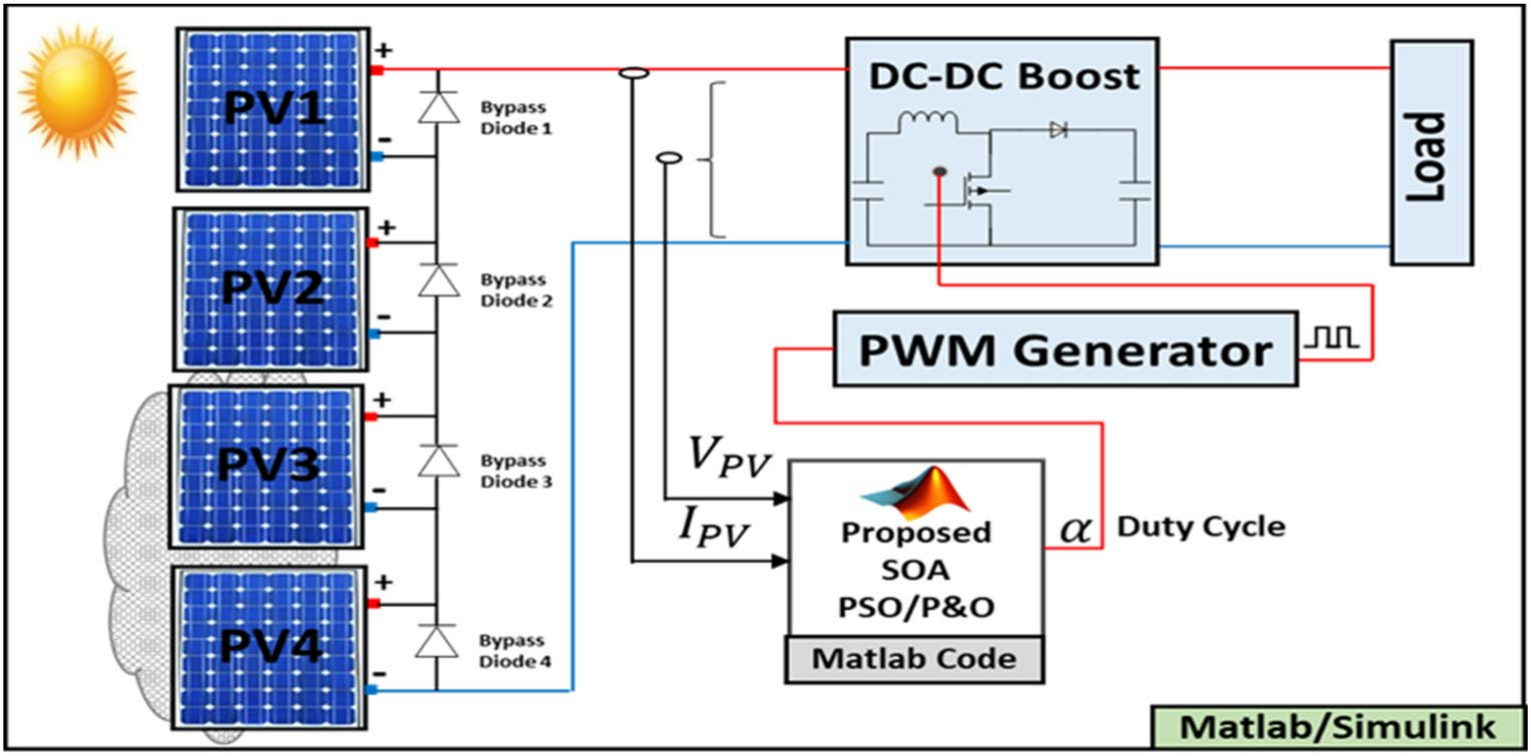
PV system configuration with the SOA based MPPT controller.

To examine the performance of the SOA based MPPT method, the 80 W PV system depicted in Fig. 7 is considered, which is designed under MATLAB/SIMULINK environment. This system comprises a PV array formed by four 20 W PV modules serially connected, a boost DC–DC converter, an MPPT controller and a DC load. The DC–DC converter is controlled, using a PWM generator, by the duty cycle "α" which is generated by the SOA based MPPT controller.

**Figure 8:**
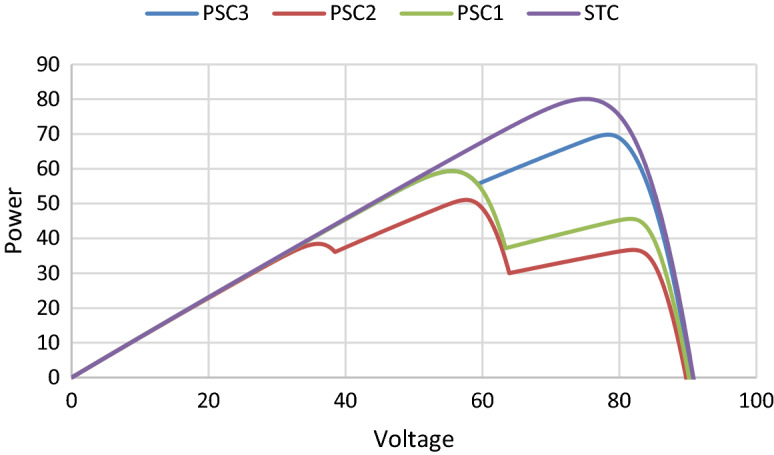
PV characteristics under different PSCs.

Table 3 shows the irradiation values considered in the simulation tests. The P–V characteristic obtained in each test is presented in Fig. 8. As can be seen in this figure, the P–V curve shows multiple peaks under PSCs. Each of these peaks is characterized by its voltage and power. The peaks number is depending on the number of shaded panels. The key parameters of the implemented MPPT methods, namely SOA, PSO and P&O, are seated in Table 4.

**Table 3:** Irradiation values parameters of the used boost converter.

Conditions	G1 (W/ m^2^)	G2 (W/m^2^)	G3 (W/ m^2^)	G4 (W/ m^2^)
STC	1000	1000	1000	1000
PSC1	500	1000	1000	1000
PSC2	400	800	1000	1000
PSC3	800	1000	1000	1000

**Table 4:** Parameters of SOA, PSO, P&O based MPPT methos.

SOA	PSO	P&O			
*Ts*	0.05	*Ts*	0.05	*Ts*	0.01
*ΔP*_*pv*_	2%	*ΔP*_*pv*_	2%	*ΔP*_*pv*_	2%
*N*_*SOA*_	6	*Np*	6	*ΔD*	0.01
*Max_it*	30	*Max_it*	30	–	–
A	[2–0]	w	0.4	–	–
*fc*	2	C1	1.6	–	–
–	–	C2	1.2	–	–

**Figure 9:**
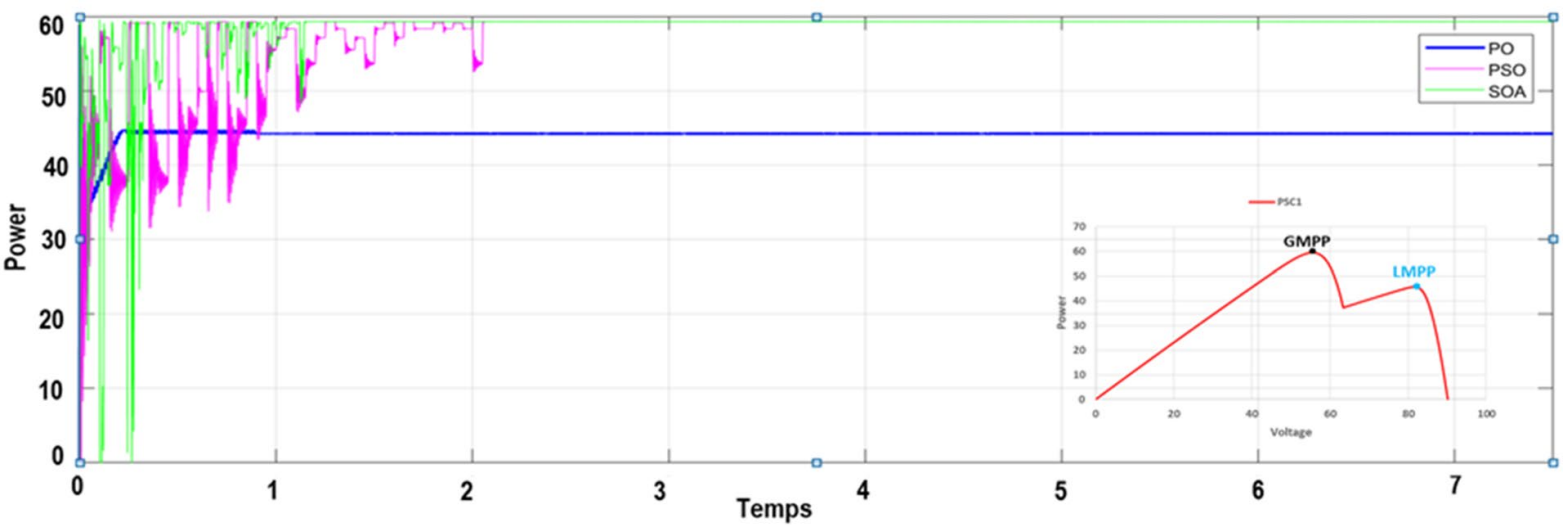
The output power obtained under PSC1 using: P&O, PSO and SOA algorithms.

**Figure 10:**
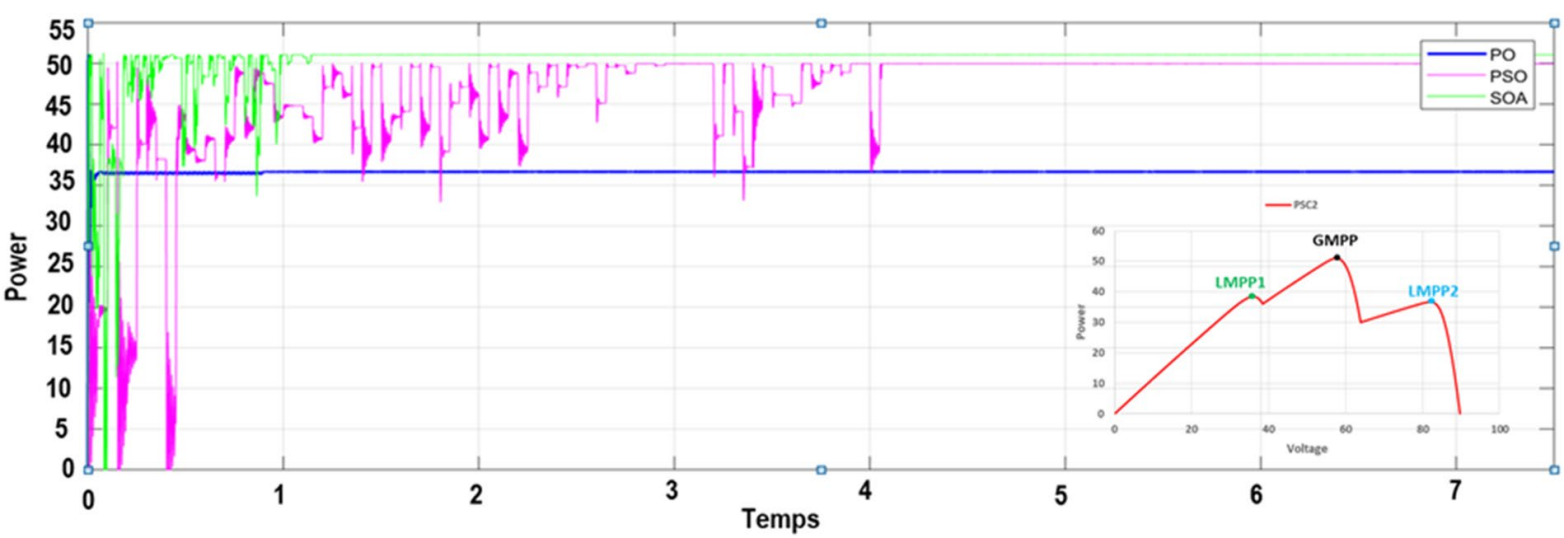
The output power obtained under PSC2 using: P&O, PSO and SOA algorithms.

**Figure 11:**
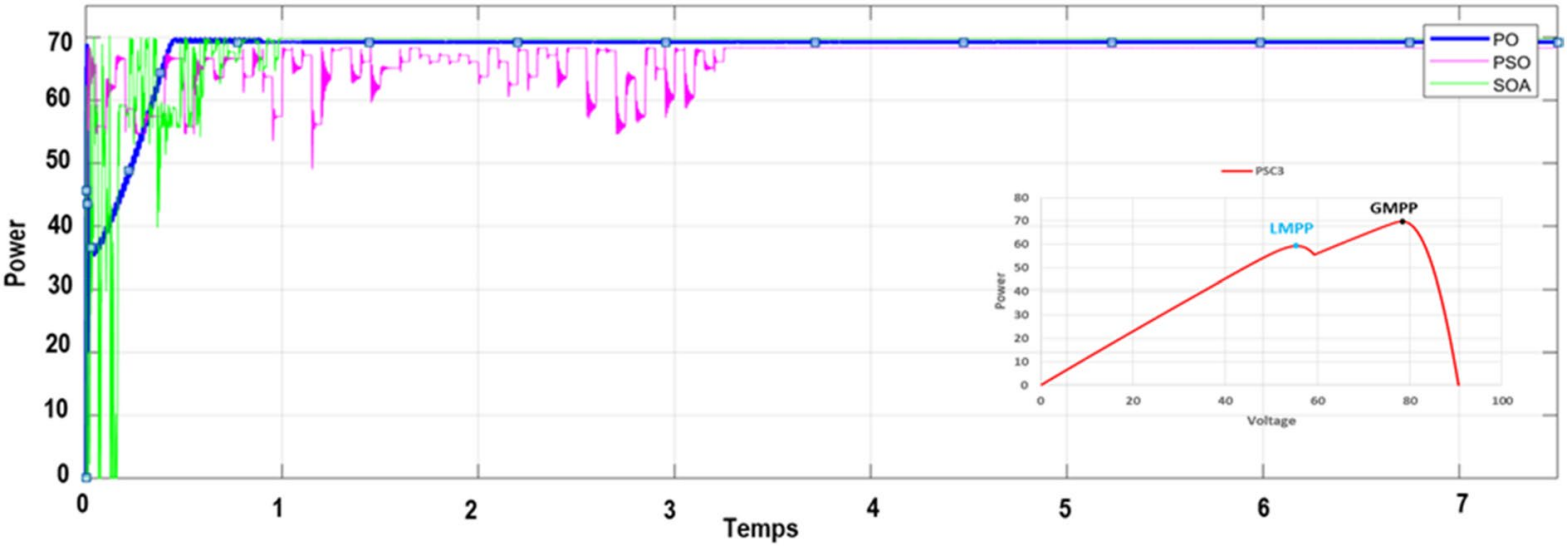
The output power obtained under PSC3 using: P&O, PSO and SOA algorithms.

**Figure 12:**
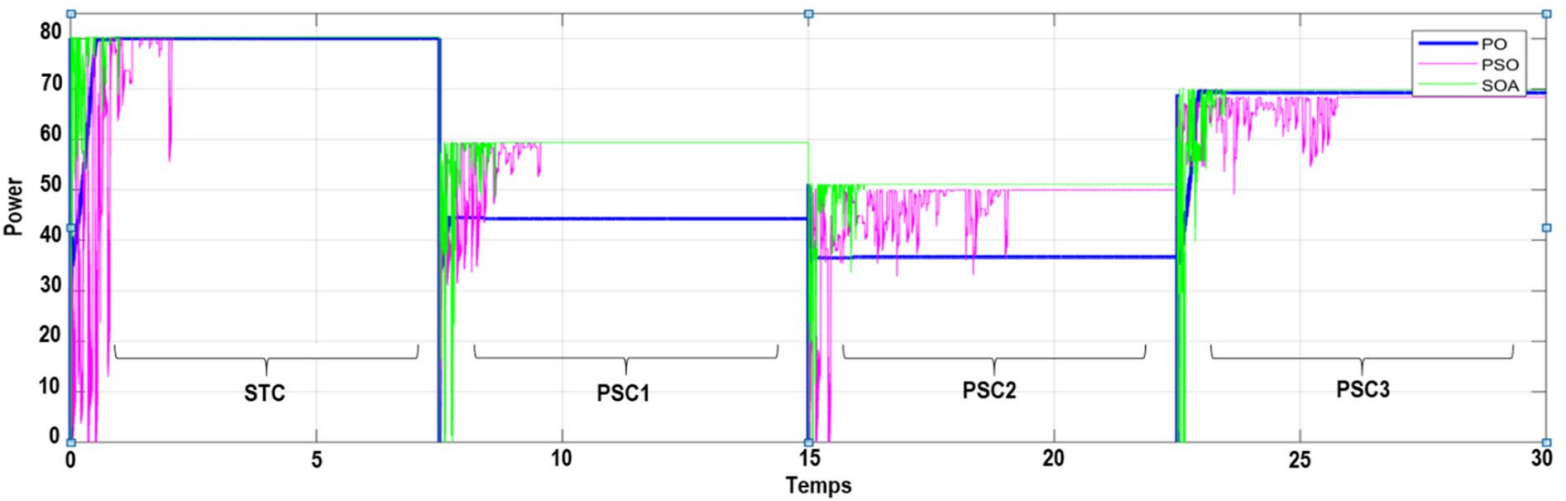
The output power obtained under STC to PSCs variation using: P&O, PSO and SOA algorithms.

To verify the ability of the SOA-based MPPT method to track the GMPP, the PV system was simulated with various PSCs (PSC1, PSC2, and PSC3) in addition to the standart test condition (STC). The system was first started by testing it under STC, and then each PSC was applied every 7.5 s for a period between 0 and 30 s, as depicted in Fig. 12. The obtained results are presented in Figs. 9,  10, 11, and 12. Under PSC 1, the SOA meets the global peak (GP) of 59.35 W with a tracking speed of 1.10 s, PSO converges to the GP of 59.30 W with a tracking speed of 2.16 s, while the P&O algorithm only converges to a local peak (LP) of 44.71 W. Under PSC 2, the SOA meets the GP of 51.06 W with 1.25 s, PSO meets the GP of 49.95 W with 4.13 s, while the P&O converges to an LP of 36.32 W. From this result, It's clear that P&O is unable to differentiate between LP and GP. In addition, when PCS 3 is applied, the SOA meets the GP of 69.79 W with 1.05 s, PSO meets the GP of 69.36 W with 3.34 s, while the P&O meets this time to the GP of 69.21 W.

Moreover, a performance comparison between SOA, PSO and P&O based MPPT methods is presented in Table 5. The obtained power in the case of the SOA-based optimization method is significantly more than those of the PSO and P&O algorithms throughout the whole profile. Table 6 presents a comparison of SOA-based MPPT versus different MPPT methods existing in the literature according to these criteria: converter used, sensors used, convergence speed, tracking eficiency, steady-state oscillations level, implementation complexity and GMPP tracking ability.

**Table 5:** Performance comparison of the SOA, PSO and P&O based MPPT methos.

	Global peak from P–V curve (W)	MPPT techniques	Maximum power (V)	Tracking time (s)	Tracking efficiency (%)
PSC1	59.86	P&O	44.71	0.85 s	73.71
		PSO	59.30	2.16 s	96.10
		Proposed MPPT–SOA	**59.35**	**1.10 s**	**97.80**
PSC2	51.36	P&O	36.32	0.92 s	71.35
		PSO	49.95	4.13 s	90.60
		Proposed MPPT–SOA	**51.06**	**1.25 s**	**98.05**
PSC3	69.95	P&O	69.21	0.75 s	97.40
		PSO	69.36	3.34 s	95.25
		Proposed MPPT–SOA	**69.79**	**1.05 s**	**97.74**
STC	80.29	P&O	80.05	0.75 s	95.80
		PSO	80.25	3.28 s	94.15
		Proposed MPPT-SOA	**80.27**	**1.09 s**	**99.25**

Significant values are in bold.

**Table 6:** Comparaison performance of SOA based MPPT with other MPPT algorithms.

MPPT algorithms	Year	Implementation complexity	Conveter type	Steady-state oscillations	Convergence speed	Sensor used	Tracking efficiency	GMPP tracking ability
P&O^9^	2015	Easy	Boost	High	Varies	I,V	Very low	No
INC^12^	2018	Easy	Boost	Low	High	I,V	Low	No
Fuzzy logic^24^	2016	High	Boost	Low	High	I,V	Medium	No
PSO^29^	2015	Medium	Boost	Medium	High	I,V	Medium	Yes
GWO^23^	2016	Medium	Boost	zero	Moderate	I,V	Medium	Yes
ABC-P&O^24^	2019	Medium	Boost	Low	Medium	I,V	High	Yes
GWO-PSO^42^	2021	Medium	Boost	Low	High	I,V	High	Yes
ACO^43^	2013	Low	Boost	High	Medium	I,V	Medium	Yes
EGWO^44^	2017	Very High	Boost	Low	High	I,V	High	Yes
BOA^45^	2019	Medium	Boost	Low	Medium	I,V	Medium	Yes
IDE^46^	2018	Medium	Sepic	Medium	High	I,V	High	Yes
MSSA^47^	2019	Medium	Boost	Low	High	I,V	Very High	Yes
L-PSO^48^	2017	Low	Boost	Medium	High	I,V	Medium	Yes
MGA-F^49^	2018	High	Buck	Medium	Medium	I,V	Medium	Yes
HGTA^50^	2018	High	Buck	Low	High	I,V,P	High	Yes
SOA Proposed	2022	Medium	Boost	Very low	Very high	I,V	Very high	Yes

**Figure 13:**
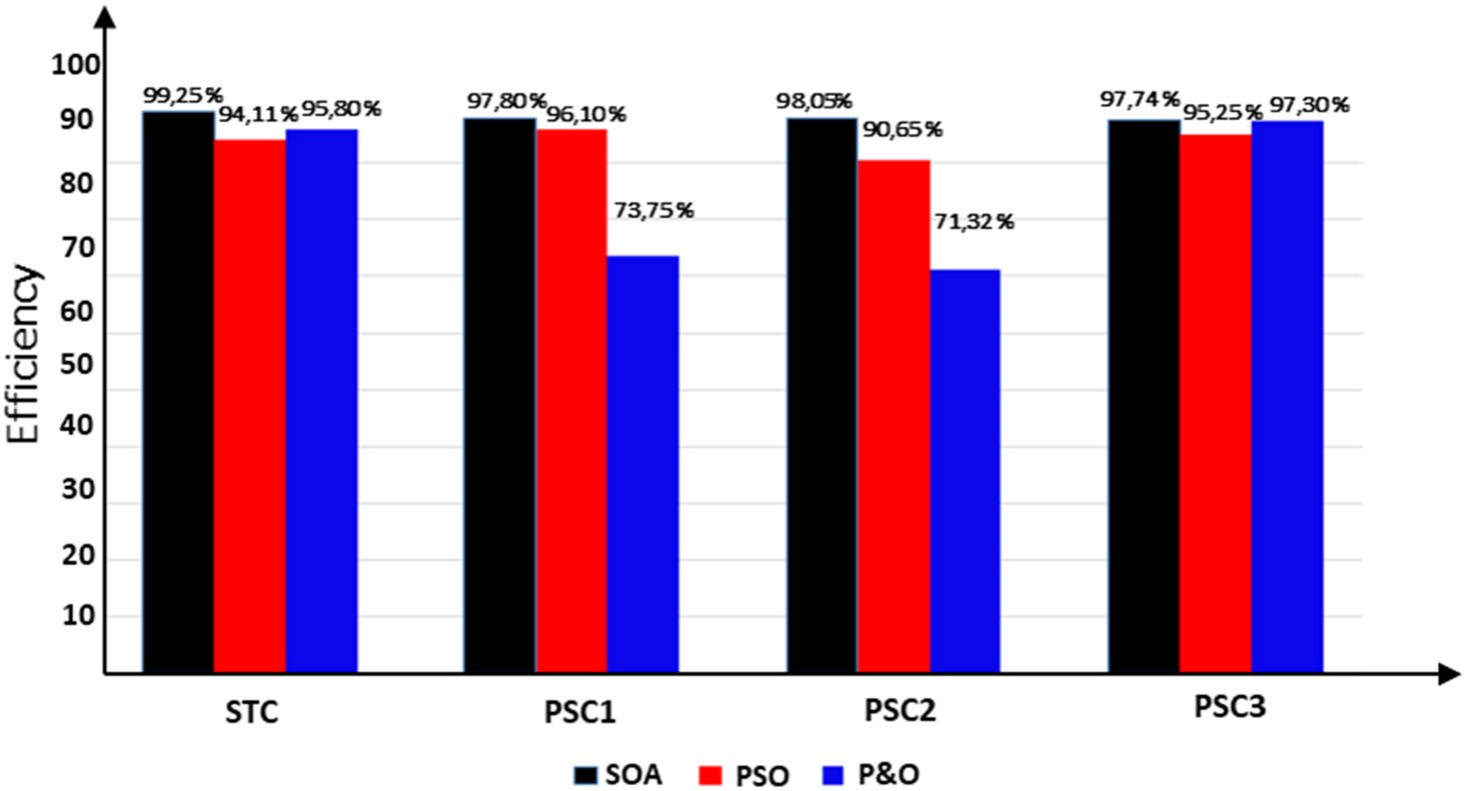
Diagram comparative of the tracking efficiency obtained using P&O, PSO and SOA methos.

Figure 13 presents the tracking efficiency of PV system output power under various PSC using P&O, PSO and SOA methods. It can be concluted that the proposed SOA-MPPT is guaranted the tracking of GMPP with high efficiency better than P&O and PSO.

Under all simulations tests, it can be observed that the the proposed SOA-MPPT and PSO algorithms successfuly converge to the GMPP corresponding to the different PSCs with a noticeable superiority of the proposed SOA-MPPT in terms tracking speed. Although the tracking speed of the P&O algorithm is higher than that of SOA and PSO. Yet, P&O algorithm is not even able to track the GMPP in the most case of PSC and is traped in the local MPP of the P–V curve (in the case of PSC1 and PSC2).

**Figure 14:**
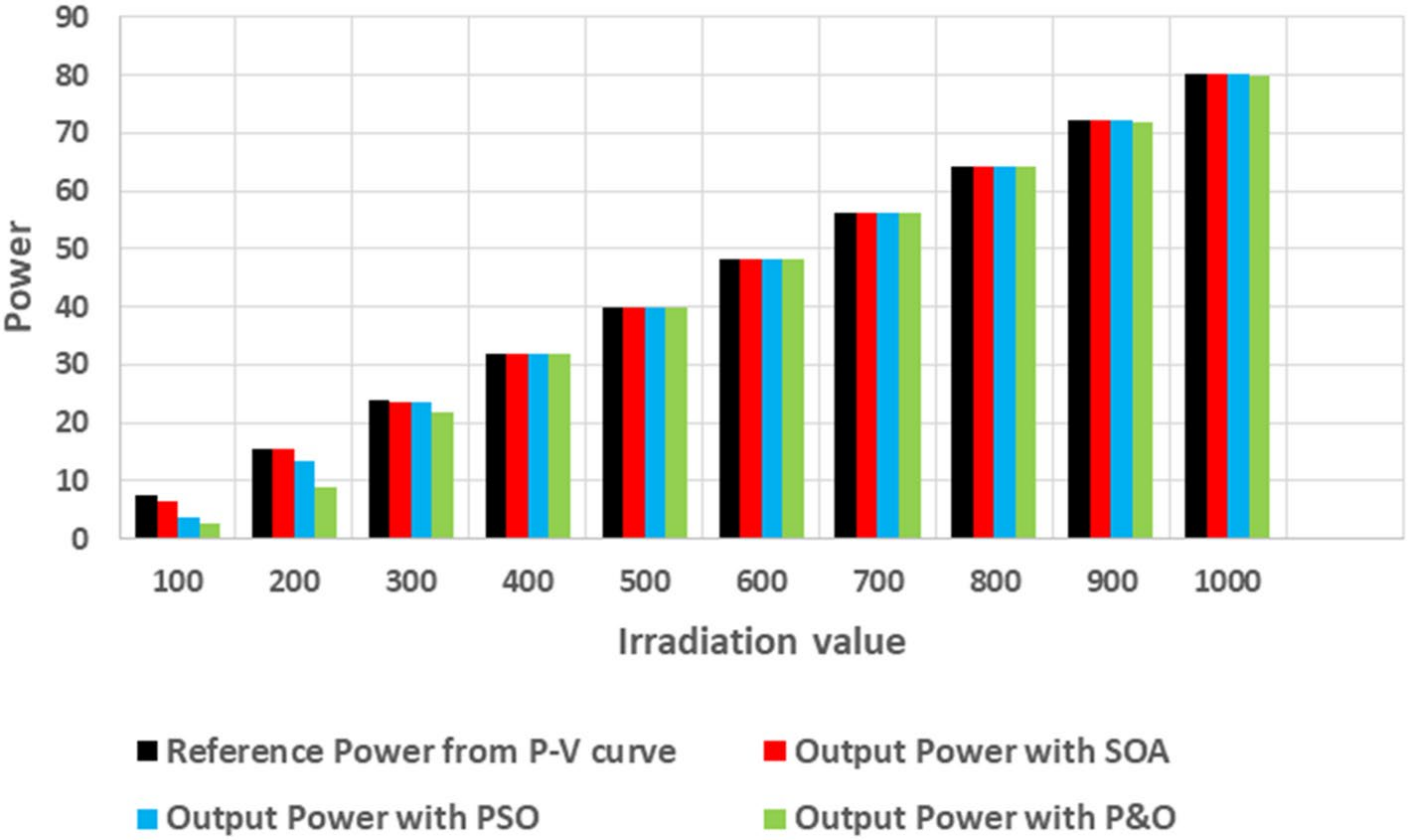
Extracted output power of PV system from different uniform irradiation using P&O, PSO and SOA methods.

In addition, we note that the P&O and PSO algorithm are not able to track the true MPP when the PV system operating under weak uniform conditions (see Fig. 14). In otherwise, the proposed SOA-MPPT successfuly converge to the MPP when the PV system operating under weak uniform conditions. Finally, it can be interpreted that the SOA based MPPT converges with a good speed and zeros oscillates around the GP compared to PSO and P&O based MPPT.

## Processor In the Loop (PIL) testing

**Figure 15:**
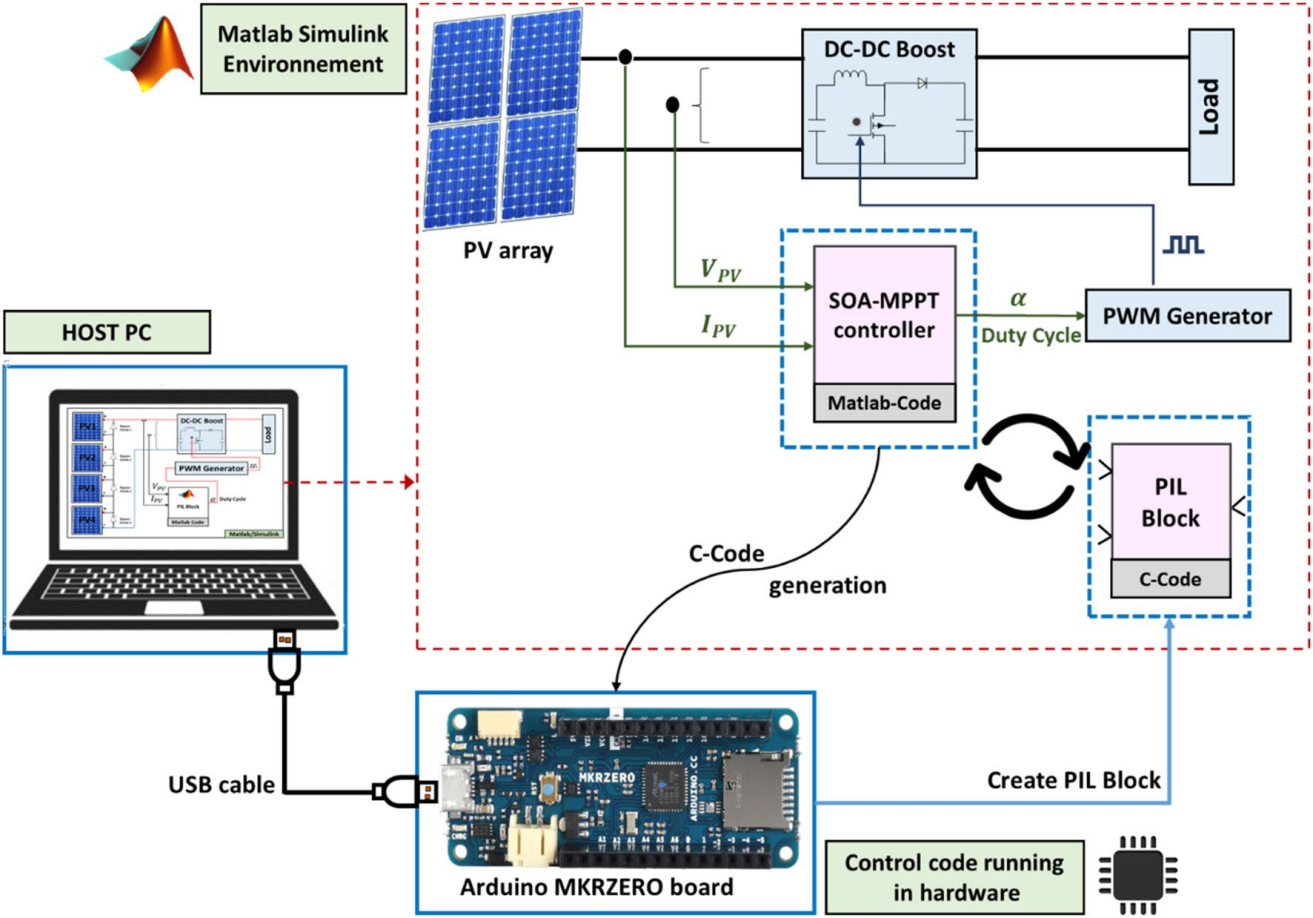
Diagram of PV generation using PIL block.

The goal of this part is to put the MPPT controller model onto a real embedded processor and run a closed-loop simulation with the simulated plant model; this is known as the processor-in-the-loop (PIL) test. In this way, the SOA-MPPT controller is replaced by a PIL block that have the controller code running on the hardware. The PIL test will help us identify if the processor is capable of executing the developed MPPT controller to validate the proposed MPPT control strategy on an actual embedded board. Figure 15 shows the embedded board used to perform the PIL experimentation, which is the Arduino MKRZERO board. The microcontroller integrated in this board is the ATMEL SAMD21 from Microchip Technology. This microcontroller contains a 32-bit Arm® Cortex®-M4F processor with Floating Point Unit (FPU) running at up to 120 MHz with 256kB flash memory, 32kB SRAM.

**Figure 16:**
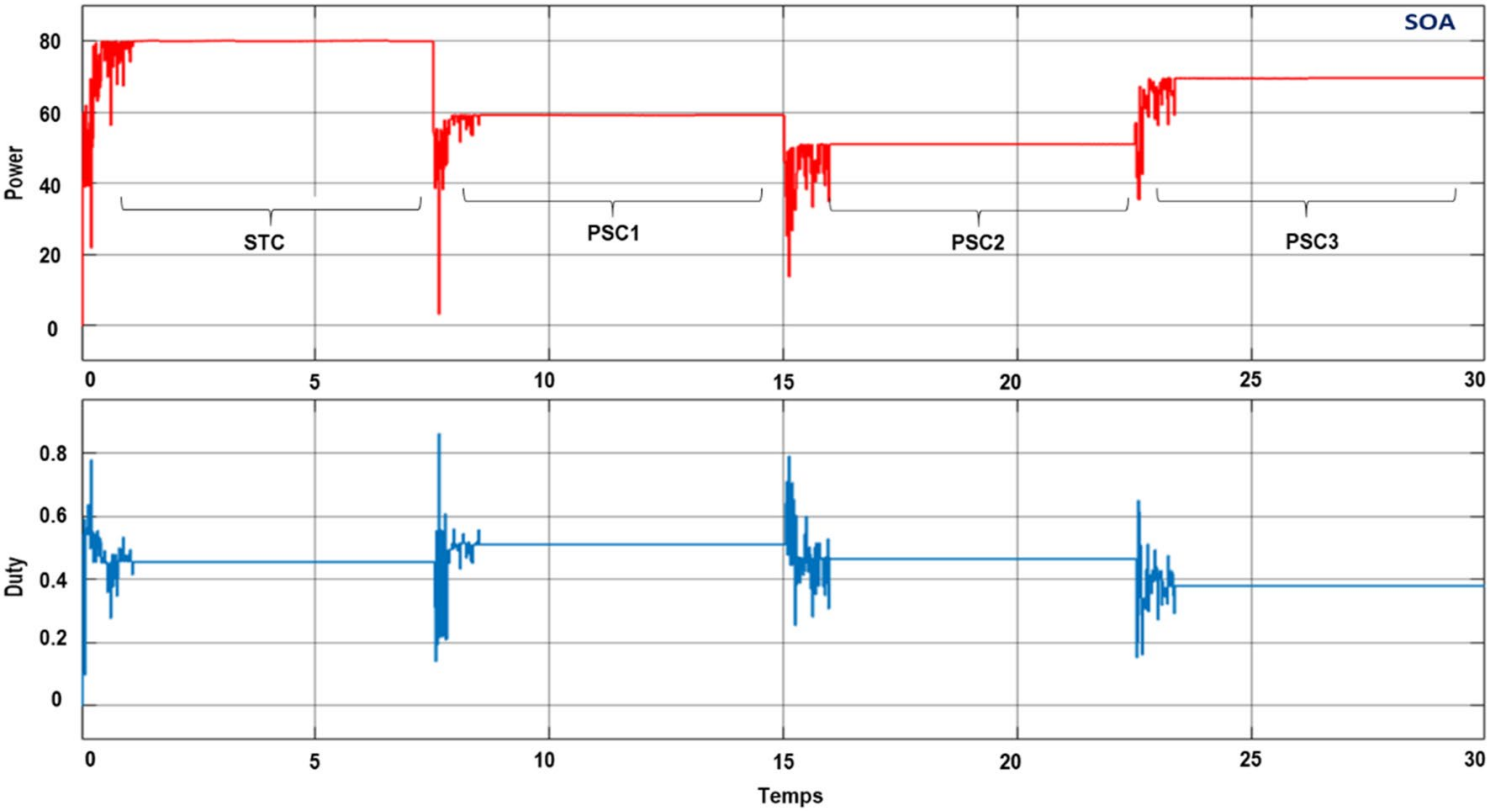
PIL results of the output power obtained under STC to PSCs variation using SOA algorithms.

As presented in Fig. 15, the PIL block is generated and connected to the plant model so as to acquire the PV output voltage and current, after that the PIL block will identify the required duty cycle by using the proposed algorithm and send it to the plant model. During the PIL process, the generated code is tested in realtime while the plant model runs on a computer which allows to detect and correct possible errors. Figure 16 depicts the result from the PIL test. It can be observed that the results obtained using PIL test are similar to the simulation results obtained in MATLAB/Simulink. Therefore, the MPPT control algorithm proposed in this work is verified on a real microcontroller (or embedded board).

## Conclusion

In this research, a new metaheuristic-based MPPT has been proposed. The letter is designed by using seagull optimization algorithm. The performance of this method is simulated and compared with that of PSO and P&O. Consequently, the effectiveness of the suggested SOA-based MPPT method was verified for an 80 W PV system using the MATLAB/SIMULINK environment. It is noted that the average tracking efficiency of the proposed method is higher than 98.32%.The simulation results were performed under various partial shading scenarios and weak uniform conditions. It prouves the superiority of the proposed method in terms of tracking efficiency and fast response time, as compared to other methods (P&O and PSO).

In this work, the design and implementation of a new MPPT method were carried out. Moreover, a Processor in the Loop (PIL) test was performed, using the Arduino MKRZERO embedded board, to confirm the functionality of the proposed SOA-MPPT approach. In this work, a PV array consisting of four modules connected in series is considered to test the proposed MPPT algorithm. However, we also aim to make complex partial shading conditions in our future research to test and confirm the effectiveness of the MPPT approach based on the proposed SOA.

## Data Availability

Corresponsdence and requests for materials should be addressed to A.C.
