# Author Correction: A novel MPPT design based on the seagull optimization algοrithm for phοtovοltaic systems operating under partial shading

**DOI:** 10.1038/s41598-023-28882-9

**Published:** 2023-02-01

**Authors:** Abdelilah Chalh, Redouane chaibi, Aboubakr El Hammoumi, Saad Motahhir, Abdelaziz El Ghzizal, Mujahed Al-Dhaifallah

**Affiliations:** 1 Innovative Technologies Laboratory, Higher School of Technologies, SMBA University, 30000 Fez, Morocco; 2 Industrial Technologies and Services Laboratory, Higher School of Technologies, SMBA University, Fez, Morocco; 3 Engineering, Systems and Applications Laboratory, ENSA, SMBA University, 30000 Fez, Morocco; 4 Control & Instrumentation Engineering Department, King Fahd University of Petroleum & Minerals, Dhahran, 31261 Kingdom of Saudi Arabia; 5 Interdisciplinary Research Center (lRC) for Renewable Energy and Power Systems, King Fahd University of Petroleum & Minerals, Dhahran, 31261 Kingdom of Saudi Arabia

Correction to: *Scientific Reports* 10.1038/s41598-022-26284-x, published online 16 December 2022

The Acknowledgements section in the original version of this Article was omitted. The Acknowledgements section now reads:

“This work was supported by the Interdisciplinary Research Center for Renewable Energy and Power Systems at King Fahd University of Petroleum & Minerals (KFUPM) under Project INRE2221.”

The original Article has been corrected.

